# Psychometric Properties of the Pain Numeric Rating Scale When Applied to Multiple Body Regions among Professional Musicians

**DOI:** 10.1371/journal.pone.0161874

**Published:** 2016-09-07

**Authors:** Mikhail Saltychev, Heidi Vastamäki, Ryan Mattie, Zachary McCormick, Martti Vastamäki, Katri Laimi

**Affiliations:** 1 Department of Physical and Rehabilitation Medicine, Turku University Hospital, Turku, Finland, and University of Turku, Turku, Finland; 2 Orton Research Institute, Orton Foundation, Helsinki, Finland; 3 Department of Orthopedic Surgery, Physical Medicine and Rehabilitation, Stanford University Hospital & Clinics, Stanford, California, United States of America; 4 The Rehabilitation Institute of Chicago/Northwestern McGaw Medical Center, Department of Physical Medicine and Rehabilitation, Chicago, Illinois, United States of America; IRCCS Istituto Auxologico Italiano, ITALY

## Abstract

**Background:**

Despite the broad popularity of a numeric rating scale (NRS) its psychometric properties are not well known. The objective was to determine if there is any difference in the discrimination ability of the NRS when used for measuring pain severity separately in different body regions.

**Methods:**

Cross-sectional survey study of 630 professional musicians. Item Response Theory (IRT) was used to define the psychometric properties of the NRS.

**Results:**

The discrimination ability of the pain NRS was dependent on the body area to which it was applied. The discrimination was low 0.5 (95% CI 0.4. to 0.7) for the hand region and perfect for the shoulder and upper part of the neck– 3.2 (95% CI 1.2 to 5.2) and 10.5 (95% CI 10.0 to 10.9), respectively. Both shoulder and neck NRSs showed a great shift towards higher levels of pain severity meaning that the ability of the NRS to discriminate low levels of pain is poor. NRS scores obtained from all other regions did not demonstrate any discrimination ability.

**Conclusions:**

The pain NRS might have different psychometric properties depending on the body area to which it is applied. Overall, the modest discrimination ability of the pain NRS implies that it should be used in screening questionnaires with some reservations.

## Introduction

The numeric rating scale (NRS) and visual analogue scale (VAS) are common measurement tools that are found in numerous pain surveys [1]. They have been used in populations with elevated levels of chronic or acute pain, as well as among generally healthy individuals. While VAS scoring is based on a continuous scale, the NRS is a typical Likert-type scale based on discrete responses. Usually, the NRS is a horizontal line with 11 marks on it–from 0 to 10 –where, measuring pain severity, 0 indicates “no pain” and 10 indicates “the worst possible pain”.

Despite the popularity of this scale, only a modest number of validation studies have been performed on the topic. Previously, the pain NRS has been found to be a reliable scale in terms of inter- or intra-rater repeatability and its ability to detect change [2–4]. Only a few research teams have used modern psychometric methods like Rasch analysis or item response theory (IRT) to investigate the validity of the pain NRS. By using Rasch analysis, Kersten et al. and Thomee et al. questioned the validity of psychometric abilities of pain VAS [5, 6]. Both of them concluded that the pain VAS does not behave linearly and its responsiveness varies along the trait of pain. Few studies used the more sophisticated method of IRT, which has been used to develop new or calibrate existing NRS-based tests [7–9].

As such, it is unclear if the pain NRS measures what it is supposed to measure, and its psychometric properties are not well known. In lieu of the non-linearity of the NRS demonstrated by previous research, the ability of the NRS to distinguish people with more pain from those with less pain is unknown. Is the NRS especially informative when applied to populations consisting of individuals with substantial pain severity, or does it more effectively differentiate people at the lower end of the scale? This knowledge may be important when developing questionnaires for populations with low as well as elevated risk of reporting pain.

The objective of the present study was to determine if there is any difference in the discrimination ability of the NRS when used to measure pain severity separately in different body regions.

## Methods

The Ethical Committee of Orton Research Institute has approved the human protocol for this anonymous questionnaire survey.

A broad questionnaire was sent by mail to 1550 orchestra musicians and students. The questionnaire contained, among others, questions on demographics, health behavior, job satisfaction, health status, and intensity of playing music. Job status was defined as studying, working, or retired. Age was defined in full years at the time of response. Intensity of playing was defined as amount of training or performing hours per week. Work years were defined in full years at the time of response. Perceived general health was defined as a score on an 11-point NRS from ‘0’–‘the worst possible health’ to ‘10’–‘the best possible health compared to the best level during a lifetime’. Perceived work ability was defined as a score on an 11-point NRS from ‘0’–‘working is impossible’ to ‘10’–‘the best work ability compared to the best level during a lifetime’. Data on pain severity during the last week was obtained using 11-point NRSs (0 to 10) regarding seven body regions: back, neck 1, neck 2, shoulder, hand, face, and jaw. The ‘overall pain’ was calculated as a maximum score on any of these seven NRSs.

In the Finnish language, there is no a specific term for the entire neck area. Instead, one word (“niska”) describes the back neck area between the base of the skull and the seventh cervical vertebra, and another word (“hartia”) describes the upper part of the trapezius muscle. In this study, the term ‘neck-A’ was used to describe the superior and the term ‘neck-B’–the area of trapezius muscle, respectively.

### Difficulty and discrimination of the pain NRS (Item Response Theory Analysis)

IRT is a modern complex statistical technique that can determine the difficulty and discrimination ability of a test [10, 11]. In the case of the pain NRS, ‘difficulty’ refers to the level of pain experienced that is needed to achieve a 0.5 probability of reporting a particular score. The difficulty shows the probability that a patient with a certain pain level will choose a corresponding NRS value that accurately represents this pain level. In an ideal situation, patients who experience an average pain level (in this particular population) should have a 0.5 probability of reporting the average NRS response of 5 points. First, the average level of a pain in the whole study population was estimated. Then, the level of pain experienced by each participant was compared to the average level of pain observed in the entire sample.

In turn, ‘discrimination’ defines how well the pain NRS distinguishes individuals who experience more pain from those who experience less pain. In this study, discrimination of 0.01 to 0.24 was considered 'none' (a totally level regression curve), 0.25 to 0.64 was considered 'low', 0.65 to 1.34 was considered 'moderate', 1.35 to 1.69 was considered 'high', and a discrimination >1.7 was considered 'perfect' (a regression curve approaching a vertical line)[10]. In the case of “a perfect test”, the steepest interval should correspond to the patients who experience the average severity of pain in the population.

IRT is invariant (sample independent), which means that one large enough sample reliably describes the entire study population and there is no need for additional sampling. It is important to recognize, though, that as information increases (information being the total number of pain scores sampled), the precision with which a score can be estimated increases as well, resulting in a decrease in standard error. It is generally accepted that a sample size of more than 500 is sufficient to analyze using IRT.

An IRT test characteristic curve is a representation of the probabilities associated with endorsement of each response to an item. Using the test characteristic curve, a test information function can be developed to characterize the amount of precision with which an item can estimate the pain level of a participant, and can be used to calculate a value for the information provided by each item. These values can then be summed across all items to form a curve that graphically shows the precision levels for an item set relative to pain levels for the sample analyzed.

### Statistical analysis

The normally distributed data were presented as means and standard deviations. The abnormally distributed data were presented as medians along with interquartile ranges (IQR) and ranges. The rating scale model (RSM) of item response theory was used. After fitting the model with the maximum amount of iterations set at 100, both parameters–difficulty and discrimination–were calculated. Results were reported along with 95% confidence intervals (95% CI) and two-tailed p-values (considering values ≤0.05 to be statistically significant). The test characteristic curve and the test information function were presented graphically. All analyses were performed using Stata/IC Statistical Software: Release 14. College Station (StataCorp LP, TX, USA).

## Results

The data were available for 630 professional musicians (response rate 41%). Sixty-two percent were working, 32% were studying, and 6% were retired (Table 1). Forty percent were playing violin or viola (Table 2). The median work history was 14 (range 0 to 48, IQR 5 to 25) years and the average duration of training or performing was 29.1 (SD 9.0, range 0 to 60) hours per week. The average body mass index was 23.7 (SD 3.5, range 16.6 to 39.7) kg/m^2^. The perceived general health and work ability were generally “good” with medians 8 and 9 out of 10 points, respectively.

**Table 1:** Basic characteristics of the sample.

Characteristics	N	Median	Min	Max	IQR^a^	
Age (years)	626	36	15	79	26	46
Work history (years)	605	14	0	48	5	25
Perceived general health (points 0–10)	630	8	1	10	7	9
Perceived work ability (points 0–10)	627	9	0	10	7	9
Pain (points 0–10)						
Back	619	1	0	9	0	3
Neck-A	617	1	0	9	0	4
Neck-B	619	2	0	9	0	4
Shoulder	620	0	0	10	0	2
Hand	618	1	0	10	0	3
Face	603	0	0	9	0	0
Jaw	604	0	0	9	0	0

^a^ Interquartile range

**Table 2:** Distribution of sample by musical instruments.

Musical instrument	Freq.	%
Violin	187	30
Viola	58	9
Cello	54	9
French horn	47	8
Double bass	41	7
Flute	37	6
Clarinet	28	4
Trumpet	26	4
Piano	26	4
Oboe	25	4
Bassoon	23	4
Percussion	22	4
Others	49	22
Total	623	100

Most of respondents experienced only mild pain (Table 1) with the median score varying from 0 to 2 on the pain NRS. The median severity of overall pain in any region was 3 (range 0 to 10, IQR 1 to 6, n = 625) on the pain NRS. The distribution of pain NRS scores is presented in Table 3.

**Table 3:** Distribution of responses on pain numeric rating scales (NRS).

NRS score	Perceived work ability		Back pain		Neck-A		Neck-B		Shoulder		Hand		Face		Jaw	
	Freq.	%	Freq.	%	Freq.	%	Freq.	%	Freq.	%	Freq.	%	Freq.	%	Freq.	%
0	8	1	300	48	228	37	201	32	347	56	286	46	527	87	493	82
1	5	1	77	12	90	15	87	14	69	11	95	15	38	6	55	9
2	9	1	60	10	82	13	89	14	54	9	62	10	13	2	20	3
3	9	1	56	9	55	9	63	10	34	5	51	8	9	1	9	1
4	7	1	27	4	34	6	47	8	23	4	24	4	1	0	5	1
5	18	3	31	5	55	9	39	6	25	4	29	5	8	1	14	2
6	26	4	31	5	27	4	30	5	15	2	30	5	3	1	3	1
7	80	13	15	2	24	4	23	4	25	4	19	3	2	0	1	0
8	135	22	16	3	17	3	34	5	16	3	16	3	1	0	3	1
9	198	32	6	1	5	1	6	1	11	2	5	1	1	0	1	0
10	132	21	none	–	none	–	none	–	1	0	1	0	none	–	none	–
Total	627	100	619	100	617	100	619	100	620	100	618	100	603	100	604	100

**Fig 1:**
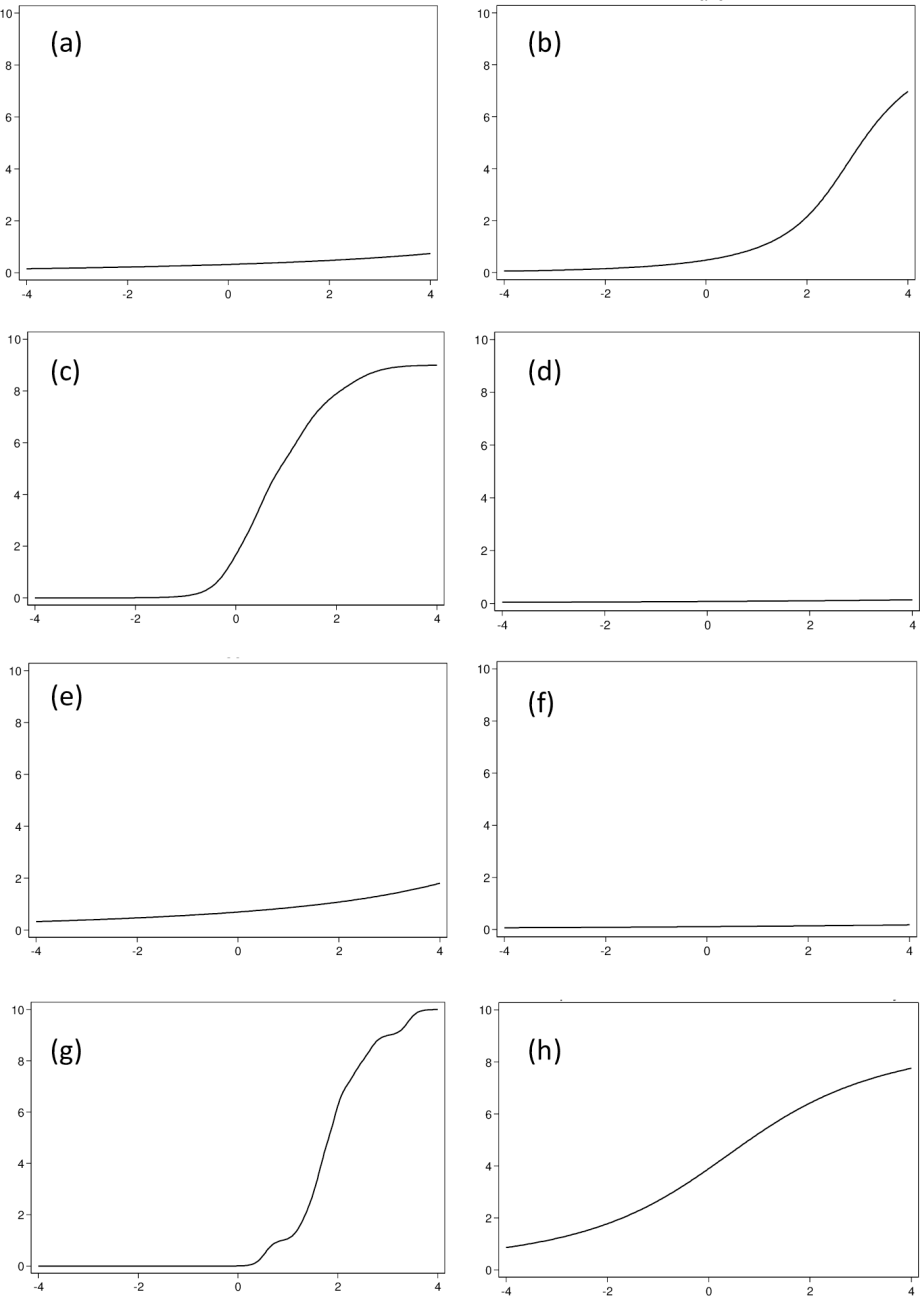
Test characteristic curves of pain numeric rating scales for different body areas. Y-axis–Expected score of numeric rating scale; X-axis–Level of pain perceived by the respondents. (a) Back, (b) Hand, (c) Neck-A, (d) Face, (e) Neck-B, (f) Jaw, (g) Shoulder, (h) Overall pain in back, neck, shoulder, hand, face, and jaw.

**Fig 2:**
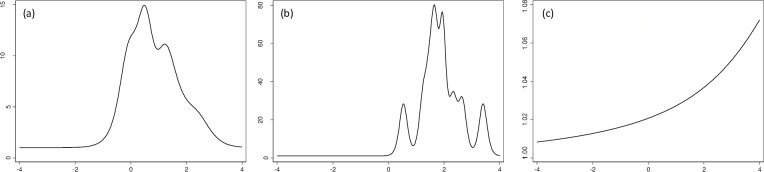
**Test information curve of numeric rating scales when applied to pain in neck-1 (left image), neck-2 (middle image), and shoulder (right image).** Y-axis–Amount of information (inverse standard error) obtainable from a test; X-axis–Level of pain perceived by the respondents.

Discrimination and difficulty results for all eight NRSs are shown in Table 4. The discrimination ability of the pain NRS was low 0.5 (95% CI 0.4. to 0.7) for the hand region and perfect for the neck-A and shoulder regions– 3.2 (95% CI 1.2 to 5.2) and 10.5 (95% CI 10.0 to 10.9), respectively. Both shoulder and neck-A NRSs showed a large shift towards higher levels of pain severity (Fig 1). This shift can also be seen in Fig 2, which indicates that the most information (inverse standard error) obtainable from the NRSs may be found at the higher levels of pain severity. NRS scores obtained from all other regions did not demonstrate any discrimination ability.

**Table 4:** Discrimination and difficulty of pain numeric rating scales used in the sample.

	Back pain			Neck-A			Neck-B			Shoulder			Hand			Face			Jaw			Pain overall		
	C^a^	95% CI		C^a^	95% CI		C^a^	95% CI		C^a^	95% CI		C^a^	95% CI		C^a^	95% CI		C^a^	95% CI		C^a^	95% CI	
Sample size	619			617			619			620			618			603			604			625		
Discrimination	0.16	0.14	0.18	3.2	1.2	5.2	0.1	-3.3	3.6	10.5	10.0	10.9	0.5	0.4	0.7	0.2	-2.9	3.2	0.1	-3.8	4.0	0.2	0.2	0.2
Difficulty																								
1 vs 0	8.4	6.3	10.4	-0.2	-0.3	0.0	5.6	-139.9	151.0	0.5	0.5	0.6	1.7	1.0	2.3	16.5	-297.0	329.9	17.3	-508.9	543.4	-0.2	-1.6	1.3
2 vs 1	9.9	8.0	11.8	-0.1	-0.3	0.1	5.3	-138.6	149.1	1.2	1.1	1.3	2.5	1.6	3.3	23.3	-414.9	461.5	25.3	-739.8	790.5	-1.0	-2.5	0.5
3 vs 2	8.9	5.5	12.3	0.3	0.1	0.5	8.0	-195.2	211.1	1.5	1.4	1.6	2.3	1.3	3.3	19.1	-332.2	370.4	23.8	-686.7	734.3	0.3	-1.8	2.4
4 vs 3	13.2	8.8	17.5	0.6	0.3	0.8	7.7	-184.1	199.5	1.6	1.5	1.7	3.6	2.3	4.9	30.7	-536.5	597.8	22.2	-634.1	678.6	1.5	-0.7	3.7
5 vs 4	7.9	4.4	11.3	0.5	0.3	0.8	7.1	-164.4	178.6	1.7	1.6	1.8	2.1	1.0	3.2	4.1	-51.1	59.3	9.7	-255.1	274.4	-1.7	-4.1	0.7
6 vs 5	8.9	5.1	12.6	1.2	0.9	1.4	7.8	-174.0	189.6	1.9	1.8	2.1	2.6	1.4	3.8	23.4	-393.2	440.0	30.0	-848.1	908.1	2.4	0.2	4.5
7 vs 6	13.6	9.5	17.7	1.3	1.0	1.5	7.9	-172.1	187.9	2.0	1.8	2.2	3.8	2.5	5.1	19.9	-325.6	365.5	26.6	-742.1	795.4	-0.6	-3.1	1.9
8 vs 7	8.7	3.4	14.1	1.7	1.4	2.0	3.5	-64.0	71.0	2.3	2.1	2.5	3.5	2.0	4.9	21.9	-355.7	399.5	9.5	-229.0	248.0	0.9	-1.5	3.3
9 vs 8	15.4	9.6	21.3	2.4	1.9	2.9	18.4	-403.5	440.2	2.7	2.3	3.0	5.5	3.4	7.7	17.7	-275.0	310.4	26.9	-735.2	789.0	4.6	1.9	7.3
10 vs 9	n/a^b^	n/a^b^	n/a^b^	n/a^b^	n/a^b^	n/a^b^	n/a^b^	n/a^b^	n/a	3.4	2.4	4.4	6.6	1.7	11.5	n/a^b^	n/a^b^	n/a^b^	n/a^b^	n/a^b^	n/a^b^	13.0	4.7	21.3

^a^ Discrimination or difficulty coefficients

^b^ not applicable

## Discussion

The results of this cross-sectional study of 630 professional musicians showed that the ability of pain NRS to differentiate people with more severe pain from those with lesser pain was good for the back region of the neck (excluding the trapezius muscle) and shoulder only. In both regions, NRS demonstrated a substantial shift towards higher levels of pain severity, meaning that the ability of NRS to distinguish between different levels of pain was inaccurate among musicians with milder pain. NRS scores obtained from all other body regions did not demonstrate any significant discrimination ability.

This was the first study to investigate the psychometric abilities of the pain NRS as a screening tool for assessing the severity of pain perceived in different body regions. This was also the first investigation on the discrimination ability of the pain NRS using IRT. The main limitation of this study is the lack of generalizability of the results. The study sample did not represent the general population but, instead, a cohort with a specific profession–professional musicians. Good general health and low levels of pain observed in the studied sample may affect the generalizability as well, even though this is unlikely due to the specific invariant nature of IRT. This study was conducted in a Finnish population and there are known cultural differences in the report and outward expression of pain.

Our results are consistent with previous reports suggesting that the relationship between the severity of pain and NRS score may not be linear [5, 6]. In addition, a study by Milojevic et al. suggested that the pain VAS (a continuous alternative of NRS) may fail when screening for severe pain[12]. We could not confirm previously apprised usefulness of the NRS in the accurate assessment of pain severity in routine clinical practice[13]. An exact comparison between our results and previous reports is not possible, as the discrimination ability of the NRS has not previously been studied using IRT. Previous studies have suggested that psychometric properties of the NRS may vary when applied to patients with different levels of cognitive or motor functioning [4, 14, 15]. The differences between the psychometric abilities of NRS when applied to pain in different body parts have not been described and the reasons for this finding are unknown. Professional musicians may perceive painful sensations in some particular body regions as “more important” than elsewhere due to specific profession-related demands. For example, a violin player may perceive back pain less restrictive then pain in the shoulder or neck–the regions under especially great stress when playing. As a result, the NRS response may be less accurate for back region and more precise for a shoulder.

Further research should address the psychometric abilities of the NRS when measuring pain severity in other body regions, the ability of the NRS to assess changes or persistence in pain over time, and the differences in these psychometric properties between populations with severe and insignificant pain. We suggest that these results to be taken into consideration when developing new pain questionnaires or when using existing surveys in clinical practice. While it has been established that the minimal clinically significant difference in pain varies by body region, the present results additionally suggest that this difference may vary along the NRS scale and it should be investigated in more detail.

### Conclusions

The present study suggests that the pain NRS may have different psychometric properties depending on the body area to which it is applied and depending on the intensity of pain reported. Overall, the modest discrimination ability of the pain NRS implies that it can be used in screening questionnaires with some reservations with regard to the population assessed.

## Supporting Information

S1 File Data underlying the findings described. (XLSX)
